# Characteristics and outcomes of ureteroscopic treatment in 2650 patients with impacted ureteral stones

**DOI:** 10.1007/s00345-017-2028-2

**Published:** 2017-03-20

**Authors:** Jaap D. Legemate, Nienke J. Wijnstok, Tadashi Matsuda, Willem Strijbos, Tibet Erdogru, Beat Roth, Hidefumi Kinoshita, Judith Palacios-Ramos, Roberto M. Scarpa, Jean J. de la Rosette

**Affiliations:** 1 Department of Urology, AMC University Hospital, Meibergdreef 9, 1105 AZ Amsterdam-Zuidoost, The Netherlands; 2 Department of Urology and Andrology, Kansai Medical University, Osaka, Japan; 3 Department of Urology, Zuyderland Medisch Centrum Parkstad, Heerlen, The Netherlands; 4 Department of Urology, Memorial Istanbul Atasehir Hospital, Istanbul, Turkey; 5 Department of Urology, University Hospital Bern, Bern, Switzerland; 6 Department of Urology, Hospital Galdakao-Usansolo, Vizcaya, Spain; 7 Department of Urology, University of Turin, Turin, Italy

**Keywords:** Urolithiasis, Impacted stones, Ureter, Ureteroscopy, Treatment outcomes, Complications

## Abstract

**Purpose:**

To describe stone-free rates and complications of ureteroscopic treatment for impacted compared with non-impacted ureteral stones and evaluate predictive variables for impaction.

**Methods:**

The Clinical Research Office of the Endourological Society prospectively collected 1 consecutive year of data from 114 centers worldwide. Patients eligible for inclusion were patients treated with ureteroscopy for ureteral stones. Patient characteristics, treatment details, and outcomes were compared with regard to stone impaction. Logistic regression analyses were conducted to explore predictive variables for ureteral stone impaction and to analyse the effect of impaction on outcomes.

**Results:**

Of the 8543 treated patients, 2650 (31%) had impacted and 5893 (69%) non-impacted stones. The stone-free rate was 87.1% for impacted stones, which is lower compared with 92.7% for non-impacted stones (*p* < 0.001). Intra-operative complication rates were higher for impacted stones (7.9 versus 3.0%, *p* < 0.001). Significantly higher ureteral perforation- and avulsion rates were reported in the impacted stone group compared with the non-impacted stone group. No association between stone impaction and post-operative complications could be shown. Female gender, ASA-score >1, prior stone treatment, positive pre-operative urine culture, and larger stones showed to be predictive variables for stone impaction.

**Conclusions:**

Ureteroscopic treatment for impacted stones is associated with lower stone-free rates and higher intra-operative complication rates compared with treatment for non-impacted stones. The predictive variables for the presence of stone impaction may contribute to the identification of stone impaction during the diagnostic process. Moreover, identification of stone impaction may aid the selection of the optimal treatment modality.

**Electronic supplementary material:**

The online version of this article (doi:10.1007/s00345-017-2028-2) contains supplementary material, which is available to authorized users.

## Introduction

Impacted ureteral stones are stones that remain unchanged at the same location for a prolonged time period causing local inflammation [1]. Confirmation of ureteral stone impaction can be done during ureteroscopy, displaying a stone enveloped in a, frequently, inflamed oedematous mucosa. The impeded stone exposure and lack of expansion space around the stone make disintegration more difficult. Patients with impacted ureteral stones are considered to be at risk for a less effective initial treatment and a higher complication rate if compared with patients with non-impacted stones [2].

For the treatment of distal and mid-ureteral stones, ureteroscopy is the approach of choice. For proximal ureter stones <10 mm, both ureteroscopy and shock wave lithotripsy (SWL) are appropriate options. Although SWL is a non-invasive modality, it is unlikely to be successful for impacted stones. For the treatment of large proximal stones, percutaneous lithotomy is an alternative, whereas laparoscopic or open lithotomy may be a rarely necessary option [3–5].

Daily practice and the literature show that the prevalence of impacted ureteral stones is high [2]. Last decades research on ureteral stone treatment has been extensive. However, the literature focused on the outcomes of ureterolithotripsy for impacted ureteral stones is limited. Furthermore, stone impaction is a neglected topic in preeminent guidelines.

The aim of this study is to compare the stone-free rate (SFR) and complication rates of ureteroscopic lithotripsy between patients with and without impacted ureteral stones and evaluate predictive variables for impaction.

## Patients and methods

### Primary objective

To assess the differences in SFR and intra- and short-term post-operative complication rates between patients treated for impacted stones compared with patients treated for non-impacted stones.

### Secondary objective

To explore variables that predict ureteral stone impaction.

### Study organization and data collection

This study examining impacted stones is a sub-analysis of the CROES URS Global Study which is a prospective, observational, international multicenter study. The CROES URS started between January 2010 and October 2011, collecting data on consecutive patients treated with ureteroscopy for urolithiasis at each participating center over a 1-year period.

114 Centers participated in 32 countries and a total of 11.885 patients were included. Data were collected and stored in the online available CROES data management system (http://www.croesoffice.org). Participating centers treated patients according to their local protocols. More detailed data containing the global URS study are described elsewhere [6].

### Study population

Patients eligible for inclusion were all patients treated with ureteroscopy for ureteral stones, as a primary or secondary treatment. Patients with ureteral stones in combination with renal stones were excluded to preclude possible influence on outcomes of renal stone treatment in the same session. Patients were divided into an impacted and a non-impacted stone group. Impaction had to be verified endoscopically [1, 2]. Whether a stone was impacted or not was determined by the surgeon and answered with the option yes or no. If stone impaction information was absent, patients were excluded from analysis.

### Patients characteristics

Patients baseline characteristics include age in years, gender, ASA-score (American Society of Anaesthesiologists score), BMI, medical history, prior stone treatment, and anticoagulant use. Stone characteristics are captured in stone width and length in mm and measured on the imaging modality that was used for pre-operative evaluation. Stone burden was calculated using the formula: length × width × 0.25 × 3.14159.

Intra-operative characteristics are operation time in minutes, intra-operative complications, and stone-free status.

### Patient follow-up and secondary treatment

Imaging consisted of x-ray and ultrasound of kidneys, ureter, and bladder, or computed tomography scan of the abdomen. Classification of a stone-free status was attained by the overall absence of stones or fragments larger than 1 mm based on imaging techniques available, and left to the discretion of the treating physician.

Post-operative characteristics, used as study outcomes, are long hospital stay (defined as longer versus shorter than 1 day), re-admission (any possible procedure related hospital visit, including re-treatment), and post-operative complications according to the Clavien–Dindo Grading system [7]. The follow-up period for the registration of post-operative complications was 3 months.

### Statistical analysis

Descriptive information is presented as mean with the standard deviation for continuous normally distributed variables, and median with inter-quartile ranges for continuous skewed variables. Categorical variables are presented as percentages. Descriptive data and percentages were based on available data. To compare outcomes between groups, Pearson’s Chi-square or Fisher’s exact test was used for dichotomous and categorical variables. A Student’s *t* test was used for continuous normally distributed variables and a Mann–Whitney *U* test for skewed variables.

Logistic regression analyses were used to determine which variables could predict stone impaction and to evaluate the association between stone impaction and outcomes after ureterolithotripsy.

Outcomes tested were SFR and intra-and post-operative complications. Outcomes were adjusted for possible confounders, including gender, ASA-score, positive pre-operative urine culture, prior stone treatment, stone burden, ureteroscopy type, difference in fragmentation device, operation time, pre-operative stent placement, and post-operative ureteral stent placement, and for differences in evaluation modalities uses to determine the absence of residual fragments.

For all analysis, the level of statistical significance was set at *p* < 0.05. Statistical analyses were performed using IBM SPSS statistics version 23.0.

## Results

### Patient characteristics

Of 11,885 patients in the URS CROES database, 8630 patients were treated for ureter stones only. Information on stone impaction was missing for 87 patients, remaining 8543 patients for analysis. 2650 (31.0%) Patients were treated for impacted stones and 5893 (69.0%) for non-impacted stones.

Table 1 presents baseline characteristics in patients with and without impacted stones. Patients with impacted stones had higher ASA II and III scores more often, higher rates of cardio vascular disease, higher rates of prior SWL treatment (20.9 versus 14.7%), and a higher stone burden. In the 6 months before, the surgery SWL in the ureteral renal unit was performed in 12.6% in the impacted stone group and 6.9% in the non-impacted stone group (*p* < 0.001). A prior ureteroscopy within 6 months before the current ureterolithotripsy was performed in 2.9% in the impacted stone group and 3.5% in the non-impacted stone group (*p* = 0.15).

**Table 1:** Baseline characteristics

Characteristics	All patients *n* = 8543	Impacted stones *n* = 2650 (31.0)	Non-impacted stones *n* = 5893 (69.0)	Difference *p* value	Type of test
Age, mean in years (SD)	47.9 (15.9) (*n* = 7328)	49.8 (15.4) (*n* = 2412)	47.0 (16.1) (*n* = 4916)	<0.001	C
Gender					
Male *n* (%)	5660 (66.3)	1867 (63.7)	3973 (67.5)	0.001	A
Female *n* (%)	2875 (33.7) (*n* = 8535)	962 (36.3) (*n* = 2649)	1913 (32.5) (*n* = 5886)		
BMI mean (SD)	26.6 (4.6) (*n* = 7147)	26.9 (4.9) (*n* = 2392)	26.4 (4.5) (*n* = 4753)	<0.001	C
ASA-score *n* (%)					
I	4795 (59.3)	1254 (49.4)	3514 (63.9)	<0.001	A
II	2670 (33.0)	982 (38.6)	1688 (30.5)		
III	587 (7.3)	288 (11.3)	299 (5.4)		
IV	31 (0.4) (*n* = 8083)	17 (0.7) (*n* = 2541)	14 (0.3) (*n* = 5542)		
Comorbidity and medication *n* (%)					
DM	840 (9.9) (*n* = 8485)	306 (11.6) (*n* = 2634)	534 (9.1) (*n* = 5851)	<0.001	A
CVD	2364 (27.7) (*n* = 8543)	910 (34.3) (*n* = 2650)	1454 (24.7) (*n* = 5893)	<0.001	A
Crohn’s disease	31 (0.4) (*n* = 8476)	11 (0.4) (*n* = 2632)	20 (0.3) (*n* = 5844)	0.59	A
Prednisone	58 (0.7) (*n* = 8479)	23 (0.9) (*n* = 2635)	35 (0.6) (*n* = 5844)	0.16	A
Anticoagulation	393 (4.6) (*n* = 8481)	166 (6.3) (*n* = 2633)	227 (3.9) (*n* = 5848)	<0.001	A
Previous stone treatment *n *(%)					
URS	880 (10.4) (*n* = 8491)	269 (10.2) (*n* = 2635)	611 (10.4) (*n* = 5856)	0.75	A
PCNL	264 (3.1) (*n* = 8494)	81 (3.1) (*n* = 2636)	183 (3.1) (*n* = 5858)	0.090	A
SWL	1407 (16.6) (*n* = 8471)	548 (20.9) (*n* = 2622)	859 (14.7) (*n* = 5849)	<0.001	A
Ureterolithotomy	90 (1.1) (*n* = 8492)	49 (1.9) (*n* = 2635)	41 (0.7) (*n* = 5857)	<0.001	A
Pyelolithomy	94 (1.1) (*n* = 8500)	37 (1.4) (*n* = 2639)	57 (1.0) (*n* = 5861)	0.080	A
Pre-operative positive urine culture *n* (%)	488 (5.9) (*n* = 8264)	214 (8.3) (*n* = 2569)	274 (4.8) (*n* = 5695)	<0.001	A
Total stone burden (mm^2^) median, [IQR]	53.4, [28–85] (*n* = 8539)	58.9, [33–95] (*n* = 2650)	50.3, [27–79] (*n* = 5889)	<0.001	D
Stone location ureter *n* (%)					
Proximal ureter	2336 (27.3)	772 (29.1)	1564 (26.5)	<0.001	A
Mid-ureter	1784 (20.9)	601 (22.7)	1183 (20.1)		
Distal ureter	4140 (48.5)	1172 (44.2)	2968 (50.4)		
Multiple locations	283 (3.3) (*n* = 8543)	105 (4.0) (*n* = 2650)	178 (3.0) (*n* = 5893)		
Ureteral stent *n* (%)	1285 (15.1) (*n* = 8496)	361 (13.7) (*n* = 2637)	924 (15.8) (*n* = 5859)	0.013	A
Percutaneous drain *n* (%)	309 (3.6) (*n* = 8498)	144 (5.5) (*n* = 2637)	165 (2.8) (*n* = 5861)	<0.001	A

*ASA* American Society of Anesthesiologists, *DM* Diabetes Mellitus, *CVD* Cardio Vascular Disease, *PCNL* Percutaneous Nephrolithotomy, *ESWL* Extra Corporeal Shockwave Lithotripsy, *URS* Ureterorenoscopy, *UPJ* uretero-pelvic junction

*NS* not significant. Data are *n* (%) of patients for whom data were available. Percentages exclude missing values from denominators. Statistical test: *A* Pearson’s Chi-square test, *B* Fishers exact test, *C* Student’s *t* test, *D* Mann–Whitney *U* test

A pre-operative ureteral stent was less often placed (13.7 versus 15.8%, *p* = 0.013) in patients with impacted stones, whereas a pre-operative percutaneous drain was placed in 5.5% in the impacted stone group and in 2.8% in the non-impacted stone group (*p* < 0.001).

### Operative characteristics

The operative characteristics, presented in Table 2, show notable higher median operation time, more frequent use of laser fragmentation, and post-operative stents in patients with impacted stones.

**Table 2:** Operation data comparing impacted stones with non-impacted stones

Outcomes	Impacted stones	Non-impacted stones	Difference *p* value	Type of test
Overall stone-free rate (SFR) *n* (%)	2293 (87.1) (*n* = 2634)	5423 (92.7) (*n* = 5850)	<0.001	A
Proximal ureter SFR *n* (%)	605 (79.3) (*n* = 763)	1364 (88.3) (*n* = 1544)	<0.001	A
Mid-ureter SFR *n* (%)	520 (86.8) (*n* = 599)	1090 (92.8) (*n* = 1174)	<0.001	A
Distal ureter SFR *n* (%)	1089 (93.2) (*n* = 1168)	2831 (95.8) (*n* = 2956)	0.001	A
Multiple ureteral locations SFR *n* (%)	79 (76.0) (*n* = 104)	138 (78.4) (*n* = 176)	0.66	A
Method of evaluation				
CT	353 (13.5)	437 (7.5)	<0.001	A
Ultrasound	1363 (52.1)	3218 (54.9)	0.017	A
X-ray/KUB	1509 (57.7)	2835 (48.4)	<0.001	A
IVU	197 (7.5)	135 (2.3)	<0.001	A
Retrograde pyelogram	24 (0.9)	138 (2.4)	<0.001	A
Intra-operative confirmation	480 (18.3)	723 (12.3)	<0.001	A
Other	30 (1.1)	33 (0.6)	0.004	A
None	132 (5.0) (*n* = 2617)	253 (4.3) *(n* = 5863)	0.14	A
Type of ureteroscope *n* (%)				
Semi-rigid	2295 (87.0)	5523 (93.9)	<0.001	A
Flexible	137 (5.2)	179 (3.0)		
Both	207 (7.8) (*n* = 2639)	181 (3.1) (*n* = 5883)		
Type of fragmentation device				
Laser	1392 (52.6)	2055 (35.0)	<0.001	A
Pneumatic	856 (32.4)	2540 (43.3)		
Other	171 (6.5)	150 (2.6)		
No device	227 (8.6) (*n* = 2646)	1121 (19.1) (*n* = 5866)		
Peroperative antibiotics *n* (%)	2313(88.0) (*n* = 2628)	4739 (80.9) (*n* = 5856)	<0.001	A
Anti-retropulsion device used	411(15.5) (*n* = 2644)	824 (14.0) (*n* = 5888)	0.062	A
Operation time minutes, median, [IQR]	40, [30–60] (*n* = 2612)	30, [21–60] (*n* = 5781)	<0.001	D
Post-operative ureteral stent *n* (%)	2333 (88.2) (*n* = 2644)	2492 (76.2) (*n* = 5893)	<0.001	A

Data are *n* (%) of patients for whom data were available. Percentages exclude missing values from denominators. *IVU* intravenous urography, *KUB* kidneys, ureters, bladder. Statistical test: *A* Pearson’s Chi-square test, *B* Fishers exact test, *C* Student’s *t* test, *D* Mann–Whitney *U* test

### Stone-free rate

The overall SFR was 87.1% for impacted ureteral stones, which is significantly lower compared with 92.7% for non-impacted stones (Table 2). For both groups, SFRs declined with an increase in stone burden (Fig. 1 supplementary material).

For the distal and proximal locations, SFR were lower with a larger burden. For the mid-ureter, SFRs were higher than 80%, even with an increased stone burden (Fig. 2 supplementary material). Stones migrated in 14.4% of the impacted stone group and in 7.9% of the non-impacted stone group. SFR for procedures were migration occurred were 52.4% for impacted stones and 86.6% for non-impacted stones.

A sub-analysis was performed to show the difference between outcomes after semi-rigid and flexible ureteroscopy for impacted stones in the proximal ureter. An ureteral access sheath was used in 61.7% of the flexible ureteroscopy cases. The SFR was 88.2% for flexible ureteroscopy and 76.4% for semi-rigid ureteroscopy, though the average stone burden was lower in the flexible ureteroscopy group. More specified results with respect to semi-rigid versus flexible ureteroscopy can be found in Table 1 in the supplementary material.

### Complications

Descriptive information on intra- and post-operative complications is shown in Table 3. The overall intra-operative complication rate was higher in patients with impacted stones. More specifically, there was a significant difference in bleeding, ureteral perforations and avulsions, unfavourable to the impacted stone group. Although there was a significantly higher overall post-operative complication rate in patients who had impacted stones, there were no differences between severity of the post-operative complications, measured by the Clavien–Dindo grades [7].

**Table 3:** Intra- and post-operative complications and outcomes

Outcomes	Impacted stones (*n* = 2650)	Non-impacted stones (*n* = 5893)	Difference *p* value	Type of test
Intra-operative complications *n* (%)				
Overall	209 (7.9)	177 (3.0)	<0.001	A
Bleeding	65 (2.5)	37 (0.6)	<0.001	A
Perforation	59 (2.2)	31 (0.5)	<0.001	A
Failed procedure	55 (2.1)	83 (1.4)	0.023	A
Conversion	7 (0.3)	5 (0.08)	0.057	B
Avulsion	9 (0.3)	1 (0.02)	<0.001	B
Other	14 (0.5) (*n* = 2643)	20 (0.3) (*n* = 5887)	0.20	A
Intra-operative migration *n* (%)	380 (14.4) (*n* = 2643)	463 (7.9) (*n* = 5887)	<0.001	A
Post-operative complications *n* (%)				
Overall	75 (2.8)	106 (1.8)	0.002	A
Bleeding	15 (0.6)	15 (0.3)	0.53	A
Fever (>38.0)	23 (0.9)	33 (0.6)	0.10	A
UTI	20 (0.8)	24 (0.4)	0.038	A
Sepsis	7 (0.3)	10 (0.2)	1.0	B
Other	10 (0.4) (*n* = 2650)	24 (0.4) (*n* = 5893)	0.84	A
Clavien grading score *n* (%)				
I	31 (40.3)	41 (38.3)	0.68	A
II	33 (42.9)	48 (44.9)		
IIIa-b	10 (13.0)	13 (12.1)		
IVa-b	3 (3.8)	3 (2.8)		
V	0 (0) (*n* = 2573)	2 (1.9) (*n* = 5786)		
Post-operative hospital stay longer than 24 h *n* (%)	1211 (45.8) (*n* = 2643)	1757 (29.9) (*n* = 5873)	<0.001	A
Re-treatment *n* (%)	307 (11.6) (*n* = 2647)	491 (8.1) (*n* = 5886)	<0.001	A
Re-admission <3 months *n* (%)	241 (9.6) (*n* = 2518)	346 (6.2) (*n* = 5615)	<0.001	A

*UTI* urinary tract infection, *NS* not significant. Data are *n* (%) of patients for whom data were available. Percentages exclude missing values from denominators. Statistical test: *A* Pearson’s Chi-square test, *B* Fishers exact test, *C* Student’s *t* test, *D* Mann–Whitney *U* test

During the follow-up period of 3 months, 11(0.4%) patients in the impacted stone group and 15 (0.3%) patients in the non-impacted stone group were re-admitted because of a ureteral stricture (*p* = 0.21).

### Post-operative outcomes

Data on length of hospital stay, re-treatment rate, and re-admission rate are shown in Table 3. Patients in the impacted stone group had a longer hospital stay, a higher re-treatment rate, and a higher re-admission rate compared with the non-impacted stone group.

### Multivariable logistic regression analysis

Logistic regression analyses, presented in Table 4, were conducted to evaluate the association between stone impaction and primary outcomes.

**Table 4:** Examining the effect of ureteral stone impaction on outcomes corrected for confounders using multivariate logistic regression

Outcomes	Odds ratio	95% confidence interval	*p* value
Model 1: Association of stone impaction and outcomes after ureteroscopy (univariate analysis)			
Stone-free rate	0.53	0.46–0.62	<0.001
Intra-operative complications	2.77	2.26–3.40	<0.001
Post-operative complications	1.59	2.65–5.98	<0.001
Model 2: Association of impaction on outcomes after ureteroscopy corrected for baseline characteristics and stone characteristics found significant in the prediction model			
Stone-free rate	0.57	0.48–0.67	<0.001
Intra-operative complications	2.71	2.17–3.38	<0.001
Post-operative complications	1.43	1.04–1.98	0.030
Model 3 Association of impaction on outcomes after ureteroscopy corrected for baseline characteristics and clinically possible confounders			
Stone-free rate	0.57	0.48–0.68	<0.001
Intra-operative complications	3.23	2.54–4.11	<0.001
Post-operative complications	1.34	0.95–1.90	0.095

Stone impaction gives an odds ratio of 0.57 on a stone-free status and an odds ratio of 3.23 on intra-operative complications. This suggests that patients with impacted stones are less often stone free, but these patients also experience more intra-operative complications. No association between ureteral stone impaction and short-term post-operative complication rate was found.

### Prediction of impaction

Table 5 shows univariate and multivariate prediction models of stone impaction. Female gender, ASA-score >1, a positive pre-operative urine culture, prior treatment and a larger stone burden showed to be predictive variables for stone impaction.

**Table 5:** Logistic regression model for predictors of stone impaction

Predictive variable	Univariate			Multivariate		
	Odds ratio	95% confidence interval	*p* value	Odds ratio	95% confidence interval	*p* value
Female gender	1.18	1.08–1.30	0.001	1.15	1.03–1.27	0.01
ASA-score						
II	1.64	1.48–1.82	<0.001	1.59	1.43–1.77	<0.001
III	2.72	2.29–3.24	<0.001	2.58	2.14–3.11	<0.001
IV	3.43	1.69–6.98	0.001	3.58	1.72–7.45	0.001
Pre-operative positive urine culture	1.87	1.55–2.26	<0.001	1.21	1.14–1.29	<0.001
Total stone burden	1.29	1.23–1.36	<0.001	1.26	1.19–1.33	<0.001
Previous treatment	1.34	1.22–1.48	<0.001	1.23	1.11–1.36	<0.001

*NS* not significant, *ASA 1 score* reference category

## Discussion

The main outcome of this study is that the treatment of impacted stones with ureterolithotripsy is associated with lower SFRs and a higher intra-operative complication rate compared with non-impacted stones. No association between stone impaction and short-term post-operative complications was found. Female gender, ASA-score >1, a positive pre-operative urine culture, prior stone treatment, and larger stone burdens were found to be predictive variables for impacted ureteral stones.

### Stone-free rate

In this study, stone impaction was associated with lower SFR. Comprehensive work analysing ureteroscopic treatment for impacted stones with the semi-rigid Ho:YAG laser was done by Seitz et al. in 2007 [2], and they report an overall SFR of 82% with an SFR of 67.2% for stones in the proximal ureter. A possible explanation for the fact that we found higher SFRs could be the expansion in use of flexible ureteroscopy, increased experience, and improved techniques in endourological surgery over the past decade [8]. Another study presented by Binbay et al. showed SFRs of 80% using a pneumatic lithotripter and 97.5% for the Ho:YAG laser. This high effectiveness can be explained by excluding patients with stones >20 mm, using permanent anticoagulants, those with ureteral strictures, multiple stones, anatomical abnormalities, renal insufficiency, and a previously unsuccessful ureteroscopic procedures [9]. In this study, we found a lower SFR for the treatment of larger (>80 mm^2^) impacted stones in the proximal ureter. The average SFR for larger stones drops to 71.4%.

Aside from the use of retrograde ureterolithotripsy, PCNL is an alternative option for the removal of larger proximal ureter stones. Previous studies compared PCNL with antegrade ureterolithotripsy for the treatment of larger (≥10 mm) impacted proximal ureteral stones. Reported SFR for PCNL varied from 96 to 100% which is higher than the SFR of 58–89% achieved with retrograde ureterolithotripsy [10–13].

The difference in SFR using ureteroscopy for impacted stones compared with non-impacted stones may be explained by several factors. The impeded stone exposure makes the surgical procedure technically more difficult [2, 14]. Furthermore, a higher intra-operative complication rate, in particular bleeding, can lead to early cessation of the procedure resulting in an incomplete stone disintegration. Moreover, in this study, stone migration also seems to influence SFRs. The prevalence of migration for impacted stones was much higher with much lower SFRs compared to migrated non-impacted stones. It could be that patients with impacted stone were more likely to have hydronephrosis or a higher degree of hydronephrosis resulting in a higher intra-operative migration rate [15].

Finally, the usage of diverse intracorporeal lithotripter devices could be a factor affecting effectiveness of ureterolithotripsy for impacted stones. In this study, this was not investigated. Previous studies revealed that the use of the Ho:YAG laser is more effective than pneumatic lithotripsy [9, 16, 17].

### Complications

Intra- and post-operative complication rates were found to be significantly related to ureteral stone impaction. The current study shows a higher intra-operative complication rate for impacted stones.

Seitz and colleagues found higher intra- and post-operative complication rates in patients with impacted stones compared to those without impacted stones. Although in both the current study and the study by Seitz, complication rates are low, the actual number may be different due to differences in patient selection and definition of complications [2]. Currently reported complication rates for ureteroscopic treatment are comparable with complication rates in overall populations [14, 18, 19].

### Predictors of stone impaction

Predictive variables could help us identify which patients have impacted stones, but may also help clarify the process of impaction. The process of stone impaction causes an inflammatory reaction of the ureteral mucosa with the genesis of oedema and fibrosis of the ureteral wall [2, 20].

We did not find literature elucidating our finding that female gender predicts impaction. Subsequently, we do not assume that there is a causal relation between female gender and impaction.

We found that patients with an ASA-score >1 are more often affected. An explanation may be the higher prevalence of comorbidity affecting the quality and regenerative capacity of ureteral tissue which can result in a higher risk of inflammation and fibrosis and thereby impaction.

Another predictor for stone impaction is prior stone treatment in the same ureteral–renal unit. We found that SWL was more often performed in patients with impacted stones. In earlier publications, it has been reported that the effect of SWL is reduced in impacted stones [1, 3, 21]. Moreover, prior SWL could increase inflammation and oedema of the ureteral wall [22]. This inflammatory reaction may contribute in the process of stone impaction. To that end, we suggest that if suspicion of impaction, SWL treatment should only be provided reluctantly and may even be contra-indicated.

We found that a positive pre-operative urine culture is a predictor for impaction. Positive urine cultures are well known as a consequence of obstructive uropathy due to impaction of ureteral stones. Moreover, infection of the urinary tract may aggravate the inflammatory reaction leading to further impaction. Whether urinary tract infections are a cause or consequence is not elucidated in this study.

Larger stones also show to be impacted more often. This is likely because stone with a larger burden have a low likelihood of spontaneous passage [3]. They get stuck in the ureter thereby causing pressure on the ureteral wall. This stone-induced ureteral wall pressure is suggested to induce ischemia, which stimulates ureteral oedema and fibrosis leading to impaction [1, 2].

The identified predictors for stone impaction may not directly be supportive to explicate preventive strategies for the reduction of stone impaction prevalence. Even so, outcomes may support future prevention strategies. Predicting characteristics contribute in the estimation which patients will be affected and help to determine the most effective and safest treatment option. However, other factors, not measured in current analyses could have predictive value. For example, a recent study by Sarica et al. showed that assessment of the acute phase reactants CRP and ESR values along with the measurement of ureteral wall thickness are predictive parameters for stone impaction [23].

### Limitations

A limitation of this study is the relatively short follow-up period of 3 months. A known complication after the treatment of impacted stones is the formation of ureteral strictures. In literature, stricture rates vary from 8 to 24% [14, 24–27]. We assume that the reported short-term stricture rate in this study firmly underestimates the long-term stricture rate. Extension of the follow-up is needed to evaluate the long-term stricture rate.

Second, we are aware that the subjectivity of defining stone impaction is an important limitation in this study. In this study, stone impaction was confirmed endoscopically. Still, the assessment was made by a large number of clinicians using their own interpretation assessing impaction.

In the literature, the definition of stone impaction is still not clarified. Most articles state an impacted stone as a stone that remains at the same location for at least 2 months. Further adduced criteria for impaction are the inability to pass the stone with a guidewire and failure to visualize contrast beyond the stone [1, 9, 28]. With the lack of one global standard, consensus on criteria to define stone impaction is suggested to optimize quality of research and compare outcomes in the literature.

## Conclusions

Ureteroscopic treatment for impacted stones showed lower SFR and higher intra-operative complication rates compared with ureteroscopic treatment for non-impacted stones. The predictive variables for the presence of stone impaction may contribute to the identification of stone impaction during the diagnostic process which may aid the selection of the optimal treatment modality.

## Electronic supplementary material

Below is the link to the electronic supplementary material.

Supplementary material 1 (DOC 35 KB)

Supplementary material 2 (TIF 48 KB)

Supplementary material 3 (TIF 55 KB)

## Abbreviations

ASA: American Society of Anesthesiologists
CROES: Clinical Research Office of the Endourological Society
BMI: Body mass index
SFR: Stone-free rate
SWL: Shock wave lithotripsy
