# Supplementary material for: Characteristics and outcomes of ureteroscopic treatment in 2650 patients with impacted ureteral stones
Source: World J Urol. 2017 Mar 20;35(10):1497–506. doi: 10.1007/s00345-017-2028-2 (PMC5613106; doi:10.1007/s00345-017-2028-2)
Supplement: Supplementary file 1 — Supplementary material 1 (DOC 35 KB) [file 345_2017_2028_MOESM1_ESM.doc]

Supplementary material

Table 1. Intra and postoperative outcomes according to type of ureteroscopy for patients who had impacted stones located in the proximal ureter.

| Type of ureteroscopy  Parameter | Semirigid (n=541) | Flexible(n=81) | Difference p-value | Type of test |
| --- | --- | --- | --- | --- |
| Stone free rate, n (%) | 410 (76.4)  (n = 537) | 67 (88.2)  (n = 76) | p = 0.018 | A |
| Stone burden(mm^2)^, median, [IQR] | 63.6, [44-115]  (n = 541) | 43.2, [16-93]  (n = 81) | p < 0.001 | D |
| Intraoperative complications n(%) |  |  |  |  |
| Overall  Bleeding  Perforation  Failed procedure | 58 (10.8)  (n = 539)  10 (1.8)  (n = 541)  16 (3.0)  (n = 541)  24 (4.4)  (n = 541) | 5 (6.2)  (n = 81)  2 (5.5)  (n = 81)  0 (0)  (n = 81)  1 (1.2)  (n = 81) | p = 0.24  p = 0.66  p = 0.25  p = 0.23 | B  B  B  B |
| Postoperative complications n(%) | 16 (3.0)  (n = 541) | 3 (3.7)  (n = 81) | p = 0.73 | B |
| Re-treatment n(%) | 114 (21.1)  (n = 540) | 10 (12.3)  (n = 81) | p = 0.074 | B |

NS = not significant(p ≥ 0.05). Data are n (%) of patients for whom data were available. Percentages exclude missing values from denominators. Statistical test: A) Pearson’s Chi-square test, B) Fishers exact test, C) Student’s t-test, D) Mann-Whitney U test.
