# Supplementary figures and images for: Characteristics and outcomes of ureteroscopic treatment in 2650 patients with impacted ureteral stones
Source: World J Urol. 2017 Mar 20;35(10):1497–506. doi: 10.1007/s00345-017-2028-2 (PMC5613106; doi:10.1007/s00345-017-2028-2)

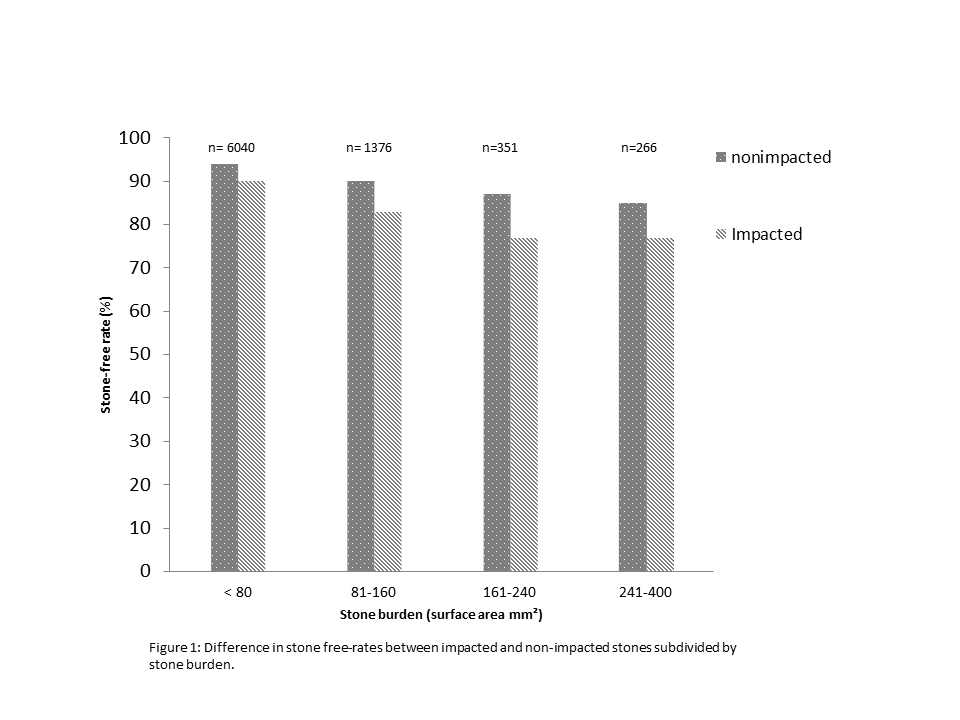

Supplement: Supplementary file 2 — Supplementary material 2 (TIF 48 KB) [file 345_2017_2028_MOESM2_ESM.tif]

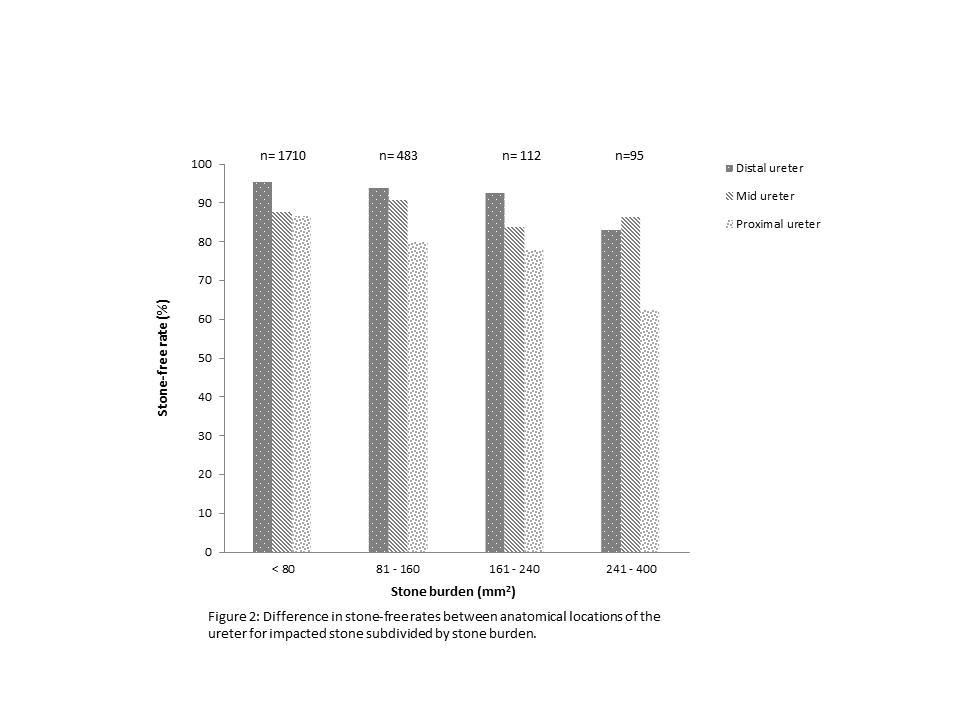

Supplement: Supplementary file 3 — Supplementary material 3 (TIF 55 KB) [file 345_2017_2028_MOESM3_ESM.tif]
